# The association between using screen in the dark and depressive symptoms: a longitudinal study among Chinese adolescents

**DOI:** 10.3389/fpsyt.2025.1618965

**Published:** 2025-08-20

**Authors:** Shuangxiao Qu, Fengyun Zhang, Xuelai Wang, Shenglei Huang, Keyang Zheng, Liting Chu, Yuting Huang, Yanting Yang, Dongling Yang, Chunyan Luo

**Affiliations:** Division of Child and Adolescent Health, Shanghai Municipal Center for Disease Control and Prevention, Shanghai, China

**Keywords:** screen time, insufficient sleep, depressive symptoms, light at night, adolescents

## Abstract

**Objective:**

To investigate the association between using screen in the dark—a predominant source of LAN—and depressive symptoms in Chinese adolescents.

**Methods:**

This study utilized a sample of 3659 adolescents (51.87% boy, mean age 14.41 ± 1.55 years) from the 2020-2022 Surveillance of Students’ Common Diseases and Health Influencing Factors in Shanghai, China. Baseline screen usage was collected by self-reported questionnaire. Depressive symptoms at baseline, 1- and 2-year follow-up were assessed using the Center for Epidemiology Scale. Generalized estimating equations were used to evaluate the association of using screen in the dark with depressive symptoms.

**Results:**

The prevalence of depressive symptoms and severe depressive symptoms in this study was 23.59% and 5.25% respectively at baseline. 15.76% adolescents who had depressive symptoms in 2020 aggravated to severe depressive symptoms in 2021 or 2022. 12.38% of the participants reported using screen in the dark. Using screen in the dark was associated with higher likelihood of depressive symptoms (*OR* = 1.89, 95%*CI*: 1.67, 2.15) and severe depressive symptoms (*OR* = 1.89, 95%*CI*: 1.54, 2.31). The adverse effect of using screen in the dark and screen time > 2 hours/day on depressive symptoms was slightly higher than only screen time > 2 hours/day (*OR* = 1.24, 95%*CI*: 1.12, 1.37) or only using screen in the dark (*OR* = 2.15, 95%*CI*: 1.73, 2.67). Additionally, those who using screen in the dark combination with screen time > 2 hours/day or insufficient sleep have the highest likelihood of depressive symptoms.

**Conclusions:**

Screen use in dark environments independently related to depressive symptoms in adolescents, with compounded associations from excessive screen time and sleep deprivation. These findings underscore the need for public health interventions targeting nighttime digital behaviors.

## 1 Introduction

Adolescent depression is increasingly recognized as a significant public health concern. According to World Health Organization, depression is estimated to occur among 1.1% of adolescents aged 10–14 years old, and 2.8% of those 15–19 years old (1). Recent research demonstrates an increasing prevalence of depression among adolescents aged 10 to 14 years in various countries (2). Furthermore, depression is one of the leading causes of illness and disability among adolescents (1). Depression can significantly affect school attendance and academic performance, exacerbate feeling of isolation and loneliness, and may ultimately lead to suicide (3). Despite these serious implications, the etiology of depression remains insufficiently understood. Multiple factors are implicated in the development of depression, including stress, bullying, harsh parenting, socioeconomic problems, disruptions to daily rhythms (4–6).

Depression is frequently accompanied by sleep disorders and circadian misalignment (7, 8). Notably, exposure to light at night (LAN) is the strongest disruptor of circadian physiology and behavior (9, 10). Technological advancements have led adolescents to devote more time to self-luminous electronic devices, including computers, smartphones, tablets, and televisions, for both educational and recreational activities. Consequently, there has been a notable increase in both the frequency and duration of electronic device usage during pre-sleep hours. A survey involving 3,749 Chinese middle school students indicated that 63.2% of 7th-grade students engage in mobile phone use while in bed (11). Comparable patterns were identified in other Asian countries, where 81.1% of Korean adolescents (12) and 25.9% of Japanese adolescents (13) reported utilizing electronic devices post-lights out. In contrast to other sources of dim ambient light, self-luminous electronic screens often emit higher levels of short-wavelength (blue) light (14). This type of light exposure is more prone to disrupting circadian rhythms and may elevate the risk of mood disorders, such as depression (4, 15).

With the widespread use of screens, numerous studies have documented the association between screen time or content and depression (16–18). However, there is a paucity of research explicitly examining the role of high levels of light at night (LAN), particularly using screen in the dark, on depressive symptoms. Understanding this relationship could inform interventions and policy-making aimed at promoting healthier lifestyles among youth.

Building on the analysis of a previous study, this research employed a dataset spanning three years (2020–2022) from the Surveillance of Students’ Common Diseases and Health Influencing Factors in Shanghai, China. The primary objective was to assess the prevalence of depressive symptoms among adolescents and to explore its correlation with the use of screens in dark environments. Additionally, the study investigated whether screen time and insufficient sleep collectively exacerbate depressive symptoms.

## 2 Materials and methods

### 2.1 Research procedures and participants

The current study was conducted as a part of the Surveillance of Students’ Common Diseases and Health Influencing Factors (SSCDHIF) in Shanghai, China, during September to November from 2020 to 2022.

**Figure 1:**
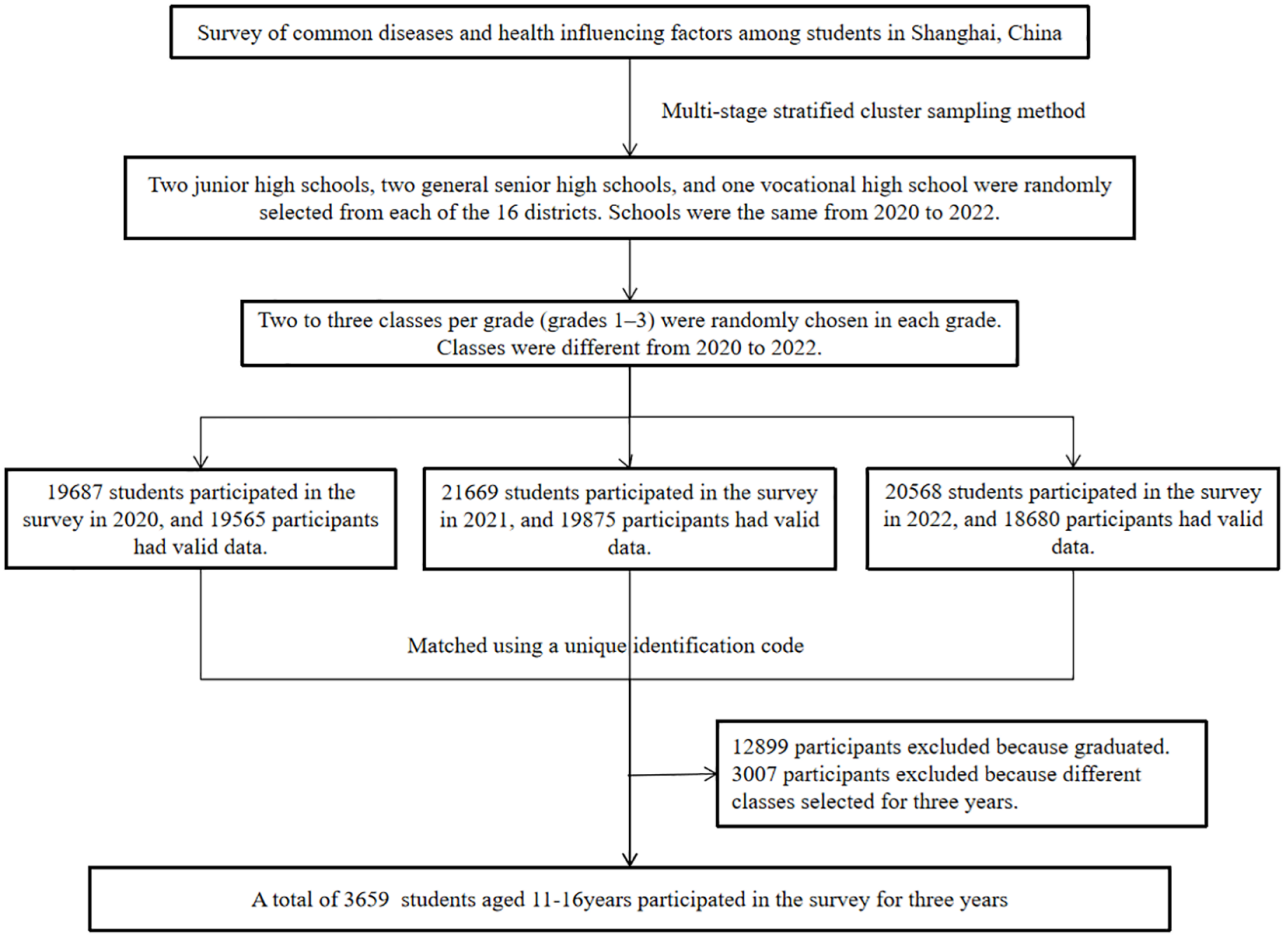
The flow chart of participant’s inclusion and matching.

A multi-stage stratified cluster sampling method was employed to create a representative sample of middle school students in Shanghai. From each of the 16 districts, two junior high schools, two general senior high schools, and one vocational high school were randomly selected. Within each selected school, two to three classes per grade (grades 1–3) were randomly chosen, and all students in these classes completed an annual questionnaire survey. 19687 students participated in the survey in 2020, and 19565 participants had valid data. 21669 students participated in the survey in 2021, and 19875 participants had valid data. 20568 students participated in the survey in 2022, and 18680 participants had valid data. After matching with a unique identification code, a total of 3659 students who participated in the surveillance program for 3 consecutive years (2020–2022) were included in this study (**Figure 1**). These students progressed from first grade in 2020 to third grade in 2022. All participants and their parents/guardians have obtained informed consent before participation. The study protocol received ethical approval from the Shanghai Municipal Center for Disease Control and Prevention Ethics Committee (Approval No. 2022-13).

### 2.2 Measurement tools

#### 2.2.1 Measurement of depression

Depressive symptoms were assessed using the Center for Epidemiologic Studies Depression Scale (CES-D), a validated instrument for assessing depressive symptomatology in adolescent populations across diverse cultural contexts (19), including demonstrated reliability in Chinese cohorts (20). The CES-D contains 20 items with 4 response options: 1) not at all or rarely (< 1 day); 2) occasionally (1–2 days); 3) often or half of the days (3–4 days); and 4) most or all of the time (5–7 days). The values of the 4 options range from 0 to 3 successively. The total score ranges from 0 to 60, with higher scores indicating higher levels of depressive symptoms. A cut-off score ≥ 16 and ≥ 28 was used to identify the depressive symptoms and severe depressive symptoms respectively (21). The Cronbach’s alpha coefficient for this survey were 0.901, 0.887, 0.866.

#### 2.2.2 Measurement of screen use in the dark

Using screen in the dark was measured as a binary variable. The original question asked respondents “Did you turn off the lights when using electronic screen after dark?” with the following response “never”, “occasionally”, “often”, “always”. Who responded “often”, “always” were recoded as 1, and who responded “never”, “occasionally” were recoded as 0.

#### 2.2.3 Measurement of screen time

Participants were asked “How long did you use electronic screen per day in the last week?”. Screen time was categorized as a binary variable with the cutoff of 2 hours/day (1= “>2 hours/day”, 0= “≤2 hours/day”).

#### 2.2.4 Measurement of sleep duration

Participants were asked “How much sleep did you get on an average day?”. According to the Health China 2030 Plan and the National Sleep Foundation’s updated sleep duration recommendations (22), adolescents who aged less than 14 years had less than 9 h of sleep on an average night; who aged 14–17 had less than 8 h of sleep; who aged over 18 had less than 7 h of sleep were considered as having an insufficient sleep, and were recoded as 1, the others had sufficient sleep were recoded as 0.

#### 2.2.5 Covariates

In this study, the sociodemographic characteristics included district, age, gender (1 = boy, 2 = girl). District was categorized as urban areas (district of Huangpu, Xuhui, Jing’an, Changning, Putuo, Hongkou, and Yangpu) and suburbs (district of Pudong, Jinshan, Minhang, Fengxian, Songjiang, Qingpu, Jiading, Baoshan, and Chongming).

We controlled for other potential risk factors which were self-reported: one-child status (1 = yes, 0 = no), family economic status (1 = good, 2 = general, 3 = poor), drinking history (1 = yes, 0 = no), academic performance (1 = good, 2 = general, 3 = poor), school bullying (1 = yes, 0 = no).

School bullying was measured based on six questions, “During the past 30 days, have you ever been bullied in the following forms? (1) Being teased in a bad way; (2) Being robed; (3) Being intentionally excluded from group activity or isolated; (4) Being threatened; (5) Being physically beaten, kicked, attacked, or squeezed; (6) Being teased of physical defects or looks”. Every question followed three responses: never, occasionally, or often. Who chose “often” for any of the six questions were categorized as having school bullying.

### 2.3 Data analysis

All the analyses were performed using R Language (V4.4.1) and SPSS 29.0. A two-tailed p value of < 0.05 was considered statistically significant. Quantitative variables were analyzed as means (standard deviation) according to the normality of distribution, and qualitative variables were analyzed as numbers (percentages). The t-test and chi-squared test were used to compare difference between sub-groups. The analytic data included depressive symptoms, using screen in the dark and other variables in each assessed interval from 2020 to 2022, and was converted to long data before Generalized Estimating Equation (GEE) analysis. Associations between using screen in the dark, screen time, sleep duration and depressive symptoms were assessed using GEE for repeated measures analysis. Model 1 was crude model. Model 2 was adjusted for district, gender, age. Model 3 was further adjusted for one-child status, family economic status, drinking history, academic performance, school bullying. The joint effect between using screen in the dark and screen time or sleep duration was also assessed.

## 3 Results

### 3.1 Participant characteristics at baseline

A total of 3659 participants were included in the analysis. Their age ranged from 11 to 19 years, and the mean age was 14.41(*SD* =1.55) years, 51.87% (1898) were boys and 48.13% (1761) were girls. The average score of CES-D was 11.06 (*SD* = 8.7). Adolescents who used screen in the dark exhibited higher CES-D scores compared to those who did not. A total of 453 participants (12.38%) reported using screens in the dark. They were more likely to have drinking history, poor family economic status, poor academic performance, insufficient sleep and screen time >2 hours/day. The demographic and behavior characteristics of adolescents at baseline are shown in **Table 1**.

**Table 1:** Baseline characteristics of adolescents.

Variables	Total (N=3659)	Using screen in the dark (N=453)	No using screen in the dark (N=3206)	*F/t*	*P*-value
Age (years), mean ± SD	14.41 ± 1.55	14.93 ± 1.42	14.34 ± 1.55	174.09	<0.001
Gender, N (%)				2.58	0.108
Boy	1898 (51.87)	219 (11.54)	1679 (88.46)		
Girl	1761 (48.13)	234 (13.29)	1527 (86.71)		
District, N (%)				0.23	0.633
Urban areas	1516 (41.43)	183 (12.07)	1333 (87.93)		
Suburbs	2143 (58.57)	270 (12.60)	1873 (87.4)		
One-child status, N (%)				3.04	0.081
Yes	843 (23.00)	119 (14.12)	724 (85.88)		
No	2816 (77.00)	334 (11.86)	2482 (88.14)		
Family economic status, N (%)				9.33	0.009
Good	1620 (44.27)	188 (11.60)	1432 (88.40)		
General	1866 (51.00)	231 (12.38)	1635 (87.62)		
Poor	173 (4.73)	34 (19.65)	139 (80.35)		
Drinking history, N (%)				88.85	<0.001
Yes	709 (19.38)	162 (22.85)	547 (77.15)		
No	2950 (80.62)	291 (9.86)	2659 (90.14)		
Academic performance, N (%)				14.33	0.001
Good	1534 (41.92)	164 (10.69)	1370 (89.31)		
General	1380 (37.72)	168 (12.17)	1212 (87.83)		
Poor	745 (20.36)	121 (16.24)	624 (83.76)		
School bullying, N (%)				3.10	0.078
Yes	67 (1.83)	13 (19.40)	54 (80.60)		
No	3592 (98.17)	440 (12.25)	3152 (87.75)		
Sleep duration, N (%)				42.73	<0.001
Sufficient	1794 (49.03)	157 (8.75)	1637 (91.25)		
Insufficient	1865 (50.97)	296 (15.87)	1569 (84.13)		
Screen time, N (%)				69.49	<0.001
Less than 2 hours/day	2752 (75.21)	269 (9.77)	2483 (90.23)		
Over 2 hours/day	907 (24.79)	184 (20.29)	723 (79.71)		
CES-D score, mean ± SD	11.06 (8.70)	15.24 (10.21)	10.47 (8.30)	33.57	<0.001

### 3.2 Prevalence of depressive symptoms among adolescents from 2020 to 2022

**Table 2** shows nearly a quarter of adolescents had depressive symptoms (CES-D score >16), and 5.25% had severe depressive symptoms (CES-D score >28) at baseline. The percentage of the participants had severe depressive symptoms increased to 5.90% in 2021 and 6.94% in 2022. The prevalence of persistent depressive symptoms and severe depressive symptoms were 7.46% and 0.74%. Furthermore, 15.76% adolescents who had depressive symptoms in 2020 aggravated to severe depressive symptoms in 2021 or 2022. Adolescents from urban areas were more likely to have depressive symptoms progression than those from suburbs. The incidence of (severe) depressive symptoms among senior high school students was higher than other students. Girls and adolescents who used screen in the dark, screen time >2 hours/day, and had insufficient sleep were more likely to have depressive symptoms in various outcomes.

**Table 2:** The prevalence of depressive symptoms in adolescents in the follow-up survey from 2020 to 2022.

Variables	Baseline depressive symptoms (N=3659)	Baseline severe depressive symptoms (N=3659)	Persistent depressive symptoms (N=3659)	Persistent severe depressive symptoms (N=3659)	Depressive symptoms cumulative incidence (N=2796)	Severe depressive symptoms cumulative incidence (N=3467)	Depressive symptoms progression (N=863)
Total, N (%)	863 (23.59)	192 (5.25)	273 (7.46)	27 (0.74)	762 (27.25)	313 (9.03)	136 (15.76)
District							
Urban areas	347 (22.89)	81 (5.34)	115 (7.59)	13 (0.86)	313 (26.78)	145 (10.10)	67 (19.31)
Suburbs	516 (24.08)	111 (5.18)	158 (7.37)	14 (0.65)	449 (27.60)	168 (8.27)	69 (13.37)
*X^2^*	0.697	0.048	0.058	0.506	0.232	3.455	5.507
*P*	0.404	0.827	0.809	0.477	0.630	0.063	0.019
Gender, N (%)							
Boys	383 (20.18)	70 (3.69)	117 (6.16)	4 (0.21)	400 (26.40)	147 (8.04)	55 (14.36)
Girls	480 (27.26)	122 (6.93)	156 (8.86)	23 (1.31)	362 (28.26)	166 (10.13)	81 (16.88)
*X^2^*	25.392	19.284	9.604	14.962	1.207	4.581	1.015
*P*	<0.001	<0.001	0.002	<0.001	0.272	0.032	0.314
School segment							
Junior high school	327 (21.89)	87 (5.82)	90 (6.02)	9 (0.60)	259 (22.19)	113 (8.03)	54 (16.51)
Senior high school	536 (24.76)	68 (5.15)	120 (9.08)	12 (0.91)	322 (32.33)	138 (11.01)	53 (16.31)
Vocational middle school	211 (25.00)	37 (4.38)	63 (7.46)	6 (0.71)	181 (28.59)	62 (7.68)	29 (13.74)
*X^2^*	4.085	2.289	9.508	0.907	28.587	9.495	0.859
*P*	0.130	0.318	0.009	0.635	<0.001	0.009	0.651
Sleep duration							
Sufficient	295 (16.44)	53 (2.95)	80 (4.46)	4 (0.22)	340 (22.68)	112 (6.43)	40 (13.56)
Insufficient	568 (30.46)	139 (7.45)	193 (10.35)	23 (1.23)	422 (32.54)	201 (11.65)	96 (16.90)
*X^2^*	99.614	37.222	45.933	12.742	34.062	28.672	1.634
*P*	<0.001	<0.001	<0.001	<0.001	<0.001	<0.001	0.201
Screen time, hours/day							
Less than 2	572 (20.78)	123 (4.47)	175 (6.36)	16 (0.58)	568 (26.06)	219 (8.33)	86 (15.03)
Over 2	291 (32.08)	69 (7.61)	98 (10.80)	11 (1.21)	194 (31.49)	94 (11.22)	50 (17.18)
*X^2^*	48.322	13.511	19.529	3.713	7.165	6.449	0.670
*P*	<0.001	<0.001	<0.001	0.054	0.007	0.011	0.413
Using screen in the dark							
No	684 (21.33)	140 (4.37)	207 (6.46)	16 (0.50)	667 (26.45)	259 (8.45)	110 (16.08)
Yes	179 (39.51)	52 (11.48)	66 (14.57)	11 (2.43)	95 (34.67)	54 (13.47)	26 (14.53)
*X^2^*	72.784	40.381	37.838	20.168	8.432	10.876	0.259
*P*	<0.001	<0.001	<0.001	<0.001	0.004	0.001	0.611

### 3.3 Associations between using screen in the dark, screen time, sleep duration and depressive symptoms

**Figure 2:**
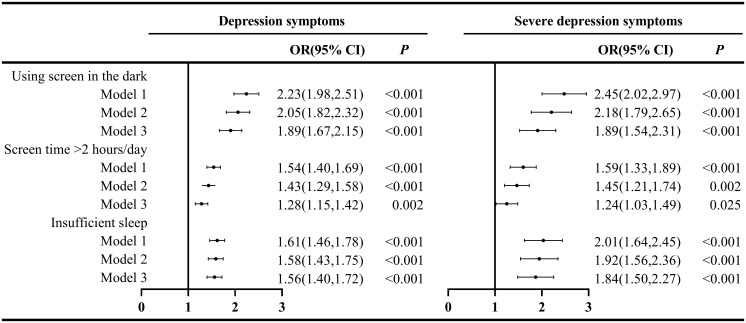
Association between using screen in the dark, screen time, sleep duration and depressive symptoms in adolescents from 2020 to 2022. Note: Model 1, crude model; Model 2, adjusted for district, gender, age; Model 3, Model 2 further adjusted for one-child status, family economic status, drinking history, academic performance, school bullying.

In the fully adjusted model, using screen in the dark was significantly associated with higher likelihood of depressive symptoms (*OR* = 1.89, 95%*CI*: 1.67, 2.15; *P* < 0.001) and severe depressive symptoms (*OR* = 1.89, 95%*CI*: 1.54, 2.31; *P* < 0.001) (**Figure 2**). Compared to screen time ≤ 2 hours/day group, screen time >2 hours/day group was associated with higher likelihood of depressive symptoms (*OR* = 1.28, 95%*CI*: 1.15, 1.42; *P* = 0.002) and severe depressive symptoms (*OR* = 1.24, 95%*CI*: 1.03, 1.49; *P* = 0.025). In addition, insufficient sleep group was associated with higher likelihood of depressive symptoms (*OR* = 1.56, 95%*CI*: 1.40, 1.72; *P* < 0.001) and severe depressive symptoms (*OR* = 1.84, 95%*CI*: 1.50, 2.27; *P* < 0.001) compared with adequate sleep group. Results from unadjusted and adjusted models were generally consistent (**Figure 2**).

**Table 3** illustrates the joint effect of using screen in the dark and screen time > 2 hours/day or insufficient sleep on depressive symptoms. We combined “using screen in the dark + insufficient sleep” and “using screen in the dark + screen time > 2 hours/day” into four groups respectively. Group 1 was the reference group, while Group 4 represented the joint effect. After adjusting for covariates, we found that *OR* of using screen in the dark and screen time >2 hours/day (*OR* = 2.22, 95%*CI*: 1.90, 2.60) on depressive symptoms was slightly higher than only screen time >2 hours/day (*OR* = 1.24, 95%*CI*: 1.12, 1.37) or only using screen in the dark (*OR* = 2.15, 95%*CI*: 1.73, 2.67). Furthermore, the *OR* for the combination of using screen in the dark and insufficient sleep (*OR* = 3.72, 95%*CI*: 2.79, 4.95) showed a substantial increase in relation to severe depressive symptoms when compared to using screen in the dark alone (*OR* = 2.09, 95%*CI*: 1.39, 3.14) or insufficient sleep alone (*OR* = 2.06, 95%*CI*: 1.65, 2.57). Likewise, the joint effects of using screen in the dark and screen time > 2 hours/day or insufficient sleep on severe depressive symptoms were consistent with depressive symptoms (**Table 3**).

**Table 3:** Joint effect of using screen in the dark and screen time > 2hours/day or insufficient sleep on depressive symptoms.

Category		Group	N (%)	Depressive symptoms		Severe depressive symptoms	
				*OR* (95% *CI*)	*P*-value	*OR* (95% *CI*)	*P*-value
Using screen in the dark	Screen time >2hours/day						
No	No	Group1	2483 (67.86)	Reference		Reference	
No	Yes	Group2	723 (19.76)	1.24 (1.12, 1.37)	<0.001	1.25 (1.15, 1.45)	0.028
Yes	No	Group3	269 (7.35)	2.15 (1.73, 2.67)	<0.001	1.93 (1.32, 2.80)	0.001
Yes	Yes	Group4	184 (5.03)	2.22 (1.90, 2.60)	<0.001	2.36 (1.82, 3.06)	<0.001
Using screen in the dark	Insufficient sleep						
No	No	Group1	1637 (44.74)	Reference		Reference	
No	Yes	Group2	1569 (42.89)	1.72 (1.54, 1.93)	<0.001	2.06 (1.65, 2.57)	<0.001
Yes	No	Group3	157 (4.29)	2.19 (1.75, 2.73)	<0.001	2.09 (1.39, 3.14)	<0.001
Yes	Yes	Group4	296 (8.09)	3.02 (2.57, 3.56)	<0.001	3.72 (2.79, 4.95)	<0.001

*CI*, confidence interval; Model adjusted for district, gender, age, one-child status, family economic status, drinking history, academic performance, school bullying.

## 4 Discussion

### 4.1 Main findings of this study

In this population-based, three-year longitudinal study conducted among 3,659 adolescents in Shanghai, China, it was found that 23.59% of participants exhibited depressive symptoms, while 5.25% experienced severe depressive symptoms in 2020. 15.76% adolescents who had depressive symptoms in 2020 aggravated to severe depressive symptoms in 2021 or 2022. Female adolescents and senior high school students were more susceptible to depression. Notably, over 10% of the adolescents reported using screens in the dark, a behavior significantly associated with depressive symptoms. Moreover, a robust association was observed between adolescents who used screens in the dark and those who experienced depressive symptoms, particularly when screen time exceeded two hours per day or when there was insufficient sleep, with the association being especially pronounced in cases of severe depressive symptoms.

The prevalence of depressive symptoms among adolescents observed in our study aligns with findings from a global meta-analysis (23), but lower than a national survey conducted at the early stage of the COVID-19 outbreak (24). The reasons might be that the epidemic has a greater impact on adolescents’ mental health, as well as the measurement instruments and cut-off scores were different in these studies. The CES-D, which was utilized in our research, is acknowledged as one of the most extensively used self-assessment tools in psychiatric epidemiology. It has also been widely applied in subsequent studies to screen for depression in adolescents. Previous studies have reported cut-off scores ranging from 14 to 30, exhibiting considerable variability without a consistent pattern (19). This variability complicates comparisons of prevalence rates across studies. Future research should focus on establishing standardized cut-off values to improve cross-study comparability.

Univariate analyses indicated that female students, screen use in dark environments, and inadequate sleep were correlated with depressive symptoms, including severe and persistent forms. High school seniors were at a heightened risk for persistent and cumulative depressive symptoms. This increased risk is attributed to the substantial academic pressure faced by high school seniors while preparing for the National College Entrance Examination, which is considered the most critical examination in their academic careers. In contrast, junior high school and vocational middle school students are exposed to comparatively lower academic demands. Consistent with previous research, our study identified a higher prevalence of depression among female adolescents (27.26%) compared to males (20.18%) in 2020. A national survey indicated that 39.5% (25) of female adolescents aged 11–18 years experienced depression, a figure exceeding that of our study, likely due to its timing during the COVID-19 outbreak in February 2020. The gender disparity in adolescent depression is a well-established phenomenon. Depression arises through multiple pathways, including biological vulnerabilities [e.g., hormonal and neurodevelopmental changes (26)] and psychosocial risk factors [e.g., low self-esteem, exposure to stress and violence (5, 27)]. Nonetheless, depression in males should not be overlooked, as the gender disparity in prevalence diminishes and stabilizes in adulthood (28). Furthermore, additional research is warranted to elucidate the genetic and social determinants underlying these gender differences in depression.

Our study identified an independent association between screen use in dark environments and depressive symptoms in adolescents. As previously mentioned, screen use in the dark may lead to increased LAN exposure. Animal studies (29–31) have demonstrated that LAN exposure can induce anti-social, anxiogenic, and depressive behaviors, even at minimal light levels as low as 5 lux. Human studies further support the LAN-depression link (32–34). A recent study confirmed that LAN is associated with mood and anxiety disorders among adolescents in the United States (4). While, the mechanisms underlying the association between screen use in dark environments and depression remain uncertain. Numerous studies have indicated that exposure to screen light during nighttime may diminish the secretion of melatonin, a hormone integral to sleep regulation, thereby disrupting the circadian timing system (35, 36). Recent research suggests that even brief exposure to light at night (0.5-3 hours) can increase levels of corticotropin-releasing hormone (CRH) and cortisol, potentially leading to heightened anxiety (37). In summary, nighttime light exposure may contribute to emotional disorders through multiple pathways: (1) dysregulation of the hypothalamic-pituitary-adrenal (HPA) axis, (2) suppression of melatonin secretion, and (3) impaired function of mood-related brain regions. Notably, adolescents show greater physiological sensitivity to LAN than adults (38, 39). The delayed melatonin rhythms and stronger suppression of melatonin by nighttime light may explain the higher prevalence of depression and anxiety in adolescents compared to adults.

More than 20% of adolescents reported engaging with electronic devices for several hours daily, a prevalence rate consistent with a study conducted in Guangzhou (6). Many studies (18, 40) have documented the adverse effects of prolonged internet use on adolescent depression. Excessive screen time has been associated with various psychological issues, including low self-esteem, stress, anxiety, depression, insecurity, and loneliness (16, 41). Several researchers have demonstrated that time spent on screens can displace participation in critical activities that may mitigate symptoms of depression, including adequate sleep (6, 42), physical exercise (43), and interpersonal communication with parents and peers. Empirical studies have confirmed the significant mediating role of these factors in the relationship between screen time and depression. Furthermore, excessive use of digital media screens may lead to various adverse effects, such as unhealthy eating behaviors, addiction, poor academic performance, cyber victimization, substance abuse, and diminished social interactions with others (44–46). These adverse effects may also contribute to mental health issues. Conversely, certain studies have indicated that internet use may alleviate depression in adolescents (47), potentially due to participation in recreational activities such as gaming, watching films or short videos, and listening to music, which can aid in stress relief and relaxation (48). Further research Future research should delineate the dual roles of screen media by jointly analyzing usage duration, content type, and contextual factors in adolescent mental health outcomes.

The current study suggests that adolescents who engage in screen use in dark environments coupled with insufficient sleep or prolonged screen time (> 2 hours/day) exhibit a higher likelihood of experiencing depressive symptoms. Notably, the combined effect of screen use in the dark and inadequate sleep significantly related to the severity of depressive symptoms. Furthermore, sleep disorders have been identified as definitive risk factors for depressive symptoms (42, 49). Numerous additional factors may indirectly influence depression by impacting sleep. A mediation analysis study indicated that electronic devices use exerted indirect effects on depressive symptoms through sleep duration reduction among adolescents (50). Nevertheless, the underlying pathophysiological mechanisms linking the combined effects of screen use in dark environments with insufficient sleep or extended screen time to depression remain inadequately understood. Additional high-quality research is required to elucidate these mechanisms.

This study has several limitations. Firstly, residual confounding may persist due to unmeasured variables such as dynamic parenting styles and fluctuating academic stress, which should be modeled as time-varying covariates in longitudinal designs. However, we adjusted for key confounders (e.g., one-child status and academic performance), strengthening the internal validity of our conclusions. Additionally, chronotype misalignment in adolescents may lead to circadian disruption, which could result in the emergence of more severe mood symptoms. It’s needed to investigate chronotype of adolescents and explore the relationship between using screen and depression. Secondly, the data utilized in this study were derived from the Surveillance of Students’ Common Diseases and Health Influencing Factors in Shanghai, China, which was not originally designed as a cohort study. We extracted data spanning from 2020 to 2022. Therefore, the representation of the samples and the resulting findings should be interpreted with caution. Because the economic level of Shanghai is higher, the results may not represent adolescents from cities with lower economic level. What’s more, the causal association and the moderate effect need to explore in the future. Thirdly, the data concerning screen use in low-light conditions were not collected with high precision. It would be advantageous to employ devices to measure screen luminance and to gather detailed information regarding screen types and the content viewed by adolescents in such conditions. The primary challenge was the large-scale nature of the survey, coupled with the fact that the participants were students, which rendered precise measurement difficult. In our future research, we aim to optimize the study design to address these limitations.

### 4.2 Conclusions

In conclusion, this longitudinal study establishes screen use in dark environments—a behaviorally modifiable source of nighttime light exposure—as an independent influencing factor for adolescent depressive symptoms. Adolescents engaging in screen use in the dark, particularly those experiencing insufficient sleep or exceeding two hours of screen time per day, exhibit a higher likelihood of developing depressive symptoms. Concurrently, there is a need for high-quality research to elucidate the causal association and its mechanisms. If these findings hold true, minimizing screen exposure in dark settings may represent a practical strategy for mitigating depression risk. It is imperative for governmental bodies to implement policy measures aimed at reducing nighttime light exposure and enhancing the mental health of adolescents. Greater attention should be directed by educational institutions and society towards addressing depression among female and senior high school students.

## Data Availability

The raw data supporting the conclusions of this article will be made available by the authors, without undue reservation.
